# Correlation between Negative Life Experiences and Auditory Hallucinations in Schizophrenia

**DOI:** 10.1192/j.eurpsy.2023.2311

**Published:** 2023-07-19

**Authors:** S. P. Godiawala, M. Parikh

**Affiliations:** 1 Psychiatry, BJ Medical College, Ahmedabad, India

## Abstract

**Introduction:**

Auditory Hallucination(AH) is a core & one of the most common symptom seen in schizophrenia. A prevalence study found that 1yr prevalence of AHs is 70% among patients of schizophrenia. Most theories of AHs discuss biological & psychological factors in phenomenology of voices. There definitely is a role of biological & neuroanatomical basis for occurrence of psychotic phenomenon & AHs, however environment & sociocultural background play important role in their genesis. It has been observed that theme of AHs is usually same, repetitive & associated with past life experiences of patients. The Negative Life Experience(NLE) though not always have significant impact on patient’s psyche, may manifest sooner or later in mental illness. Although AHs may be comforting, helping to cope up with unacceptable unconscious conflict, most patients report them to be negative, distressing & affecting socio-occupational functioning, thus making it important to understand & treat them effectively. Often, AHs stays as a residual symptom with which patient has to deal throughout life. This study is an effort to bridge existing gaps for better understanding of AHs & hence, Schizophrenia. It aims to understand effect of NLE on AHs, & thereby contribute to aetiology & therapeutic services to reduce distress of patients.

**Objectives:**

This study aims to find presence of history of past NLE in patients with schizophrenia having AHs, find a correlation between NLE & the content of AHs & hence to establish the role of sociocultural factors in phenomenology of AH in patients with schizophrenia.

**Methods:**

A longitudinal study of 30 patients diagnosed with Schizophrenia as per DSM-5 having AHs visiting Psychiatry Department of Government Hospital was carried out over a period of 2yrs. A quantitative study to find correlation between presence of NLE & AH in patients and a qualitative study by in-depth interview of 10 patients with help of semi-structured questionnaire to understand phenomenology by procedures of interpretative phenomenological analysis was carried out.

**Results:**

A positive statistically significant correlation between NLE & content of AH was found. Majority of patients experienced AHs based on NLE in past & heard voices related to it. Various themes such as Guilt, Fear, Inadequacy, Anger, Frustration surfaced during the qualitative study.

**Conclusions:**

This study thus strengthens the holistic model where sociocultural factors with biological & psychological factors play a role in pathogenesis of AHs. Treatment weighing on addressing NLE & emotions attached to it may help in foreshortening recovery & deal with residual AHs. This can help in therapeutic management of distress in patients, relieve internal conflict within their psyche, where they would feel more understood and learn to live with persistent AHs.

**Disclosure of Interest:**

None Declared

